# Electrostatic features for nucleocapsid proteins of SARS-CoV and SARS-CoV-2

**DOI:** 10.3934/mbe.2021120

**Published:** 2021-03-09

**Authors:** Wenhan Guo, Yixin Xie, Alan E Lopez-Hernandez, Shengjie Sun, Lin Li

**Affiliations:** 1 Computational Science Program, University of Texas at El Paso, El Paso, TX 79968, USA; 2 Department of Physics, University of Texas at El Paso, El Paso, TX 79968, USA

**Keywords:** SARS-CoV, SARS-CoV-2, COVID-19, protein-protein interactions, protein-RNA/DNA interactions, electrostatic force, DelPhi, DelPhiForce

## Abstract

COVID-19 is increasingly affecting human health and global economy. Understanding the fundamental mechanisms of Severe Acute Respiratory Syndrome CoronaVirus 2 (SARS-CoV-2) is highly demanded to develop treatments for COVID-19. SARS-CoV and SARS-CoV-2 share 92.06% identity in their N protein RBDs’ sequences, which results in very similar structures. However, the SARS-CoV-2 is more easily to spread. Utilizing multi-scale computational approaches, this work studied the fundamental mechanisms of the nucleocapsid (N) proteins of SARS-CoV and SARS-CoV-2, including their stabilities and binding strengths with RNAs at different pH values. Electrostatic potential on the surfaces of N proteins show that both the N proteins of SARS-CoV and SARS-CoV-2 have dominantly positive potential to attract RNAs. The binding forces between SARS-CoV N protein and RNAs at different distances are similar to that of SARS-CoV-2, both in directions and magnitudes. The electric filed lines between N proteins and RNAs are also similar for both SARS-CoV and SARS-CoV-2. The folding energy and binding energy dependence on pH revealed that the best environment for N proteins to perform their functions with RNAs is the weak acidic environment.

## 1. Introduction

Severe Acute Respiratory Syndrome CoronaVirus 2 (SARS-CoV-2) is currently affecting human health and global economy seriously. A similar situation happened in 2003 with SARS-CoV, which also belongs to Coronavirus family. SARS-CoV and SARS-CoV-2 share 92.06% identity in their N protein RBDs’ sequences [1,2]. Both SARS-CoV and SARS-CoV-2’s genomes encode nonstructural replicase polyproteins and structural proteins [1], including the nucleocapsid phosphoprotein (N protein). The main function of N protein is to link envelopes to the +RNA. The N protein of SARS had shown to play a crucial role in regulating viral RNA synthesis in replication and transcription [3]. Understanding the fundamental mechanisms of how N proteins Receptor Binding Domains (RBDs) of SARS-CoV and SARS-CoV-2 bind RNAs is highly demanded for developing new antiviral drugs and vaccines [4].

Some groups studied the N proteins of SARS-CoV and SARS-CoV-2 using experimental methods. Sisi Kang et al. [3] utilized chemical experiments and X-ray analysis to obtain the structure of N protein of SARS-CoV-2, which helped revealing potential drug targeting sites. Veverka V’s laboratory [5] performed NMR-based titration experiments, combined with computational model, to build the complex model of the Nucleocapsid N-Terminal RNA binding Domains (N-NTD) with RNA. Unfortunately, only a few groups have conducted research on the structure and function of N proteins of SARS-CoV. Peter Kuhn’s team [6] is one of them who characterized the structures of the N-NTD of SARS-CoV. Compared with experimental studies, some effort has been also made to investigate SARS-CoV and SARS-CoV-2 using computational approaches. Most of these computational studies focused on the spike (S) proteins of the SARS-CoV and SARS-CoV-2 [7,8], including discoveries of potential drug targets for SARS-CoV-2 [9-11], few works focused on N proteins. Some studies calculated the electrostatic potential on N protein surfaces in coronavirus [3,5,6]. Electrostatic features of N proteins help us understanding different mechanisms of RNA recognition and assembly. Other calculations between N proteins and RNAs explore more fundamental principles for their binding mechanisms.

Due to the relatively high cost of experiments and the rapid development of computational algorithms [12,13], computational methods are now widely used to study biology phenomena, including biomolecular structures [14,15], biomolecular interactions [16-18], pH dependence of protein-protein/DNA/RNA interactions [19,20], etc. Using such state of art computing techniques, a lot of efforts have been contributed to study viruses [7,21,22]. In this work, several computational approaches are used to study nucleocapsid proteins of SARS-CoV and SARS-CoV-2, including DelPhi [23], DelPhiForce [24,25], DelPhiPKa [26,27]. The electrostatic features are critical in analyzing the interactions between the N protein and RNA. Thus, the electrostatic potential, electric field lines and electrostatic forces were analyzed based on the structures of N proteins of SARS-CoV/SARS-CoV-2 RBDs and RNAs. It was found that SARS-CoV and SARS-CoV-2 have similar electrostatic potential distributions on their binding surfaces, which demonstrated that the net charges play a significant role to attract the RNAs. In addition, DelPhiPKa was implemented to calculate the binding energy pH dependence. Such method has been proved successful and reliable [20,28-30]. The pH effects on the binding energies for N proteins’ RBDs interacting with RNAs and folding energies of N proteins was analyzed, which demonstrated the optimal pH for N proteins’ folding and binding with RNAs. Such details assist us to understand how the N proteins’ RBDs recognize RNAs. These findings pave the way for research on future coronavirus-caused diseases. No experimental studies have been conducted to reveal the differences between the biology functions of SARS-CoV and SARS-CoV-2. Therefore, this work of comparing the N proteins of SARS-CoV and SARS-CoV-2 can also be useful for future experimental design.

## 2. Methods

### 2.1. Structure preparation

The complex structure of SARS-CoV-2 with the Double Strand RNA (dsRNA) was obtained from Protein Data Bank (pdb ID: 7ACS [5]). The SARS-CoV structure was obtained from Protein Data Bank (pdb ID: 2OFZ [6]), which does not include the dsRNA structure. Therefore, the complex structure of dsRNA combined with SARS-CoV N protein was modeled by aligning the SARS-CoV structure to SARS-CoV-2 based on the template of 7ACS using Chimera [31]. This study is mainly focused on the electrostatic features of Nucleocapsid N-Terminal RNA binding Domains (N-NTDs) of SARS-CoV and SARS-CoV-2. In the SARS-CoV N protein structure, the N and C terminals are not determined [6]. Figure S1 shows the complex structures of SARS-CoV-2 N protein RBD binding with RNA, which is determined by NMR experiments [5]. The NMR structures demonstrate none of the N or C terminals of SARS-CoV-2 binds to RNAs, therefore the N and C terminals are extremely flexible. Due to this experimental evidence, N and C terminals of SARS-CoV-2 were deleted in this work. After the deletion, we obtained the same length of N proteins for SARS-CoV-2 and SARS-CoV.

### 2.2. Electrostatic Calculations using DelPhi and DelPhiForce

DelPhi [23] and DelPhiForce [24,25] tools focus on accurate calculations and visualizations of the electrostatic potential and forces for biomolecules. They were used to calculate the electrostatic potential and total force for the N protein RBD and RNA binding domain. Finite difference (FD) method is implemented in the DelPhi and DelPhiForce tools to solve the Poisson-Boltzmann equation (PBE):

(1) $$\nabla \cdot [\epsilon (r)\nabla \phi (r)]=-4\pi \rho (r)+\epsilon (r)\kappa^{2}(r)\sinh(\phi (r)∕k_{B}T),$$

Where ф(r) is the electrostatic potential, ε(r) is the dielectric permittivity, ρ(r) is the permanent charge density according to the atomic structure, κ is the Debye–Huckel parameter, *k*_*B*_ is the Boltzmann constant, and T is temperature.

**Figure 1.**
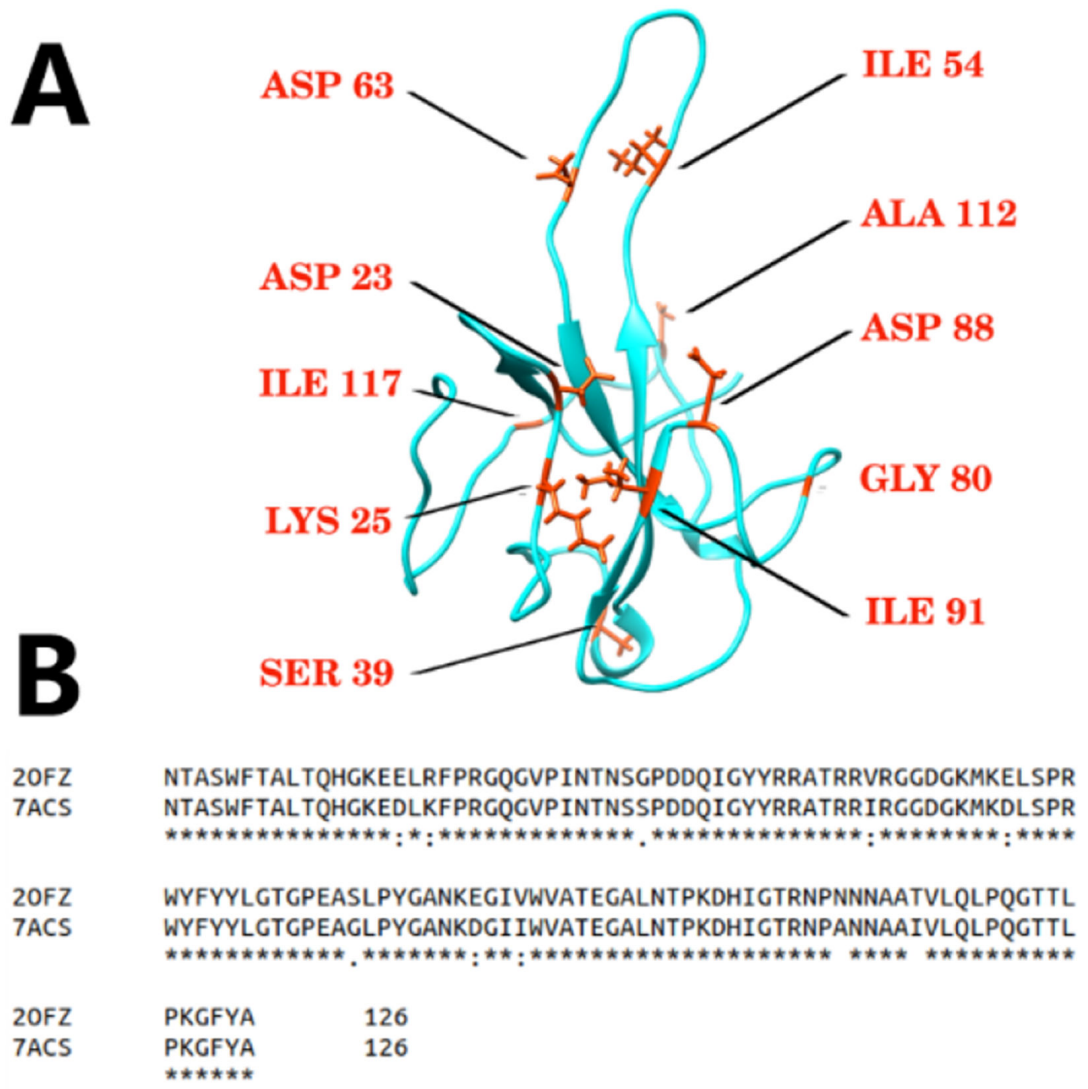
Mutations on SARS-CoV-2 compared with SARS-CoV. A. The structure of SARS-CoV-2 N proteins. The mutation sites are highlighted in orange color. B. Sequence alignment of N proteins of SARS-CoV and SARS-CoV-2.

The electrostatic potential of the SARS-CoV N protein and SARS-CoV-2 N protein with RNA domain was calculated by DelPhi. Their surfaces were visualized by Chimera [31] using the color scale range from −1.0 to 1.0 kT/e (see Figure 1). In order to compare the directions and strengths of electrostatic forces, the N protein and RNA was separated from 5 Å to 40 Å with the step size of 5 Å using StructureMan [32]. Then at each position, the electrostatic force was calculated by DelPhiForce. The visual molecular dynamics (VMD) [33] was implemented to visualize the total forces and the electric field lines between N protein and RNA.

### 2.3. Folding energy calculation methods

DelPhiPKa [26,27] was implemented to calculate pKa values of nucleotides of RNAs’ and proteins’ ionizable residues. The net charge of the unfolded state was calculated with the following equation:

(2) $$Q_{u}(pH)=\sum_{i=1}^{N}\frac{10^{-2.3y(i)(pH-pKa(i))}}{1+10^{-2.3y(i)(pH-pKa(i))}},$$

where the summation is all titratable groups, y(i) is −1 for acidic groups and +1 for basic groups.

The pKa values of SARS-CoV N protein and SARS-CoV-2 N protein with RNA domain were calculated by DelPhiPKa. The pKa range was set from 0 to 14 with an interval of 0.5 in the calculations. The pH-dependence of the folding free energy using the equation:

(3) $$\Delta N(pH_{folding})=2.3\text{RT}\int_{pH_{i}}^{pH_{f}}(Q_{f}(pH)-Q_{u}(pH)d(pH)),$$

Where *Q_f_*(*pH*) and *Q*_*u*_(*pH*) are the total net charge of folded and unfolded states. R is the universal gas constant taken as 1.9872 × 10^−3^ $\frac{kcal}{Mol*K}$. T is the temperature, which is 300 K.

### 2.4. Binding energy calculation methods

The pH-dependence of the binding energy of N proteins with RNAs was modeled by obtaining the pH-dependence of the net charge of the complexes and their components. The pH dependence of the stability of the complexes and their components using the equation:

(4) $$\Delta N(pH_{binding})=2.3RT\int_{pH_{i}}^{pH_{f}}(Q_{t}(pH)-Q_{n}(pH)-Q_{r}(pH))d(pH)$$

where Δ*N*(*pH*_*binding*_) is the pH-dependence of the binding free energy, *Q*_*t*_(*pH*), *Q*_*n*_(*pH*), and *Q*_*r*_(*pH*) are the net charges of complex, N protein and RNA, R is the universal gas constant taken as 1.9872 × 10^−3^ $\frac{kcal}{Mol*K}$ . T is the temperature, which is 300 K.

## 3. Results and discussion

There are no experimental studies which investigated the differences between the biology functions of SARS-CoV and SARS-CoV-2. Therefore, this work which compared the N proteins of SARS-CoV and SARS-CoV-2 can be used for future experimental design. The mutations between SARS-CoV and SARS-CoV-2 N proteins were analyzed to show the distribution of mutations on the SARS-CoV-2 N protein. Furthermore, the electrostatic features of N proteins of SARS-CoV and SARS-CoV-2 were investigated. Finally, the binding and folding energies of the complexes and their components were calculated and analyzed.

### 3.1. Mutations between SARS-CoV and SARS-CoV-2

The structures of N protein RBDs of SARS-CoV and SARS-CoV-2 are very similar (the RMSD is 0.967Å). We aligned the sequences of N protein RBDs of SARS-CoV and SARS-CoV-2 using clustal omega [34] to analyze their sequence differences. The positions of the mutation sites are marked in Figure 1 with orange color. Most of the mutation sites in the N protein RBD are distributed on or closed to the hairpin-like structure. It suggests that the flexibilities of the hairpin-like structure N protein RBDs may be different between these two viruses. The flexibility of the SARS-CoV-2 N protein’s hairpin-like loop structure is shown in Figure S1.

### 3.2. Electrostatic potential on surfaces

**Figure 2.**
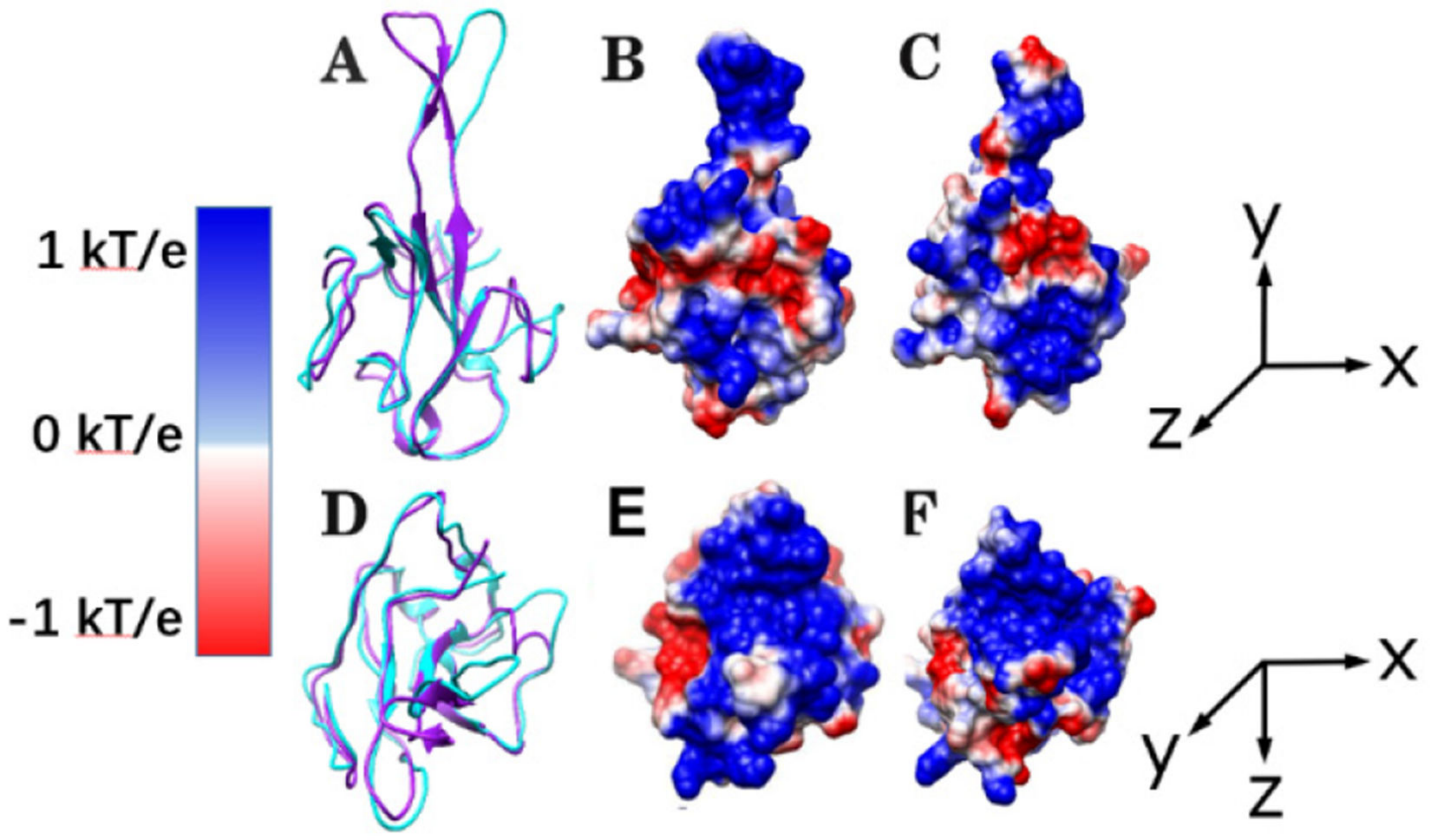
Structure and electrostatic surfaces of N proteins of SARS-CoV/SARS-CoV-2. (A) The side view of the structure of SARS-CoV (purple) and SARS-CoV-2 (cyan) N proteins. (B) The side view of the electrostatic potential on the surface of SARS-CoV; (C) The side view of the electrostatic potential on the surface of SARS-CoV-2; (D) The top view of the structure of SARS-CoV (purple) and SARS-CoV-2 (cyan) N proteins; (E) The top view of the electrostatic potential on the surface of SARS-CoV; (F) The top view of the electrostatic potential on the surface of SARS-CoV-2.

The electrostatic features are important for protein structure and functions. We calculated the electrostatic potential of the N proteins of SARS-CoV and SARS-CoV-2. With the analysis, the binding interfaces of N proteins showed dominantly positive electrostatic potential (see Figure 2) while the RNAs are negatively charged. Thus, the N protein RBDs are attracted by RNAs because the two interfaces have opposite net charges. Such a phenomenon is common in the interactions between biomolecules [7,35]. The electrostatic features of N proteins and RNAs indicate that the electrostatic binding forces between N proteins and RNAs may enhance the stabilities of the complexes.

### 3.3. Electric field lines

**Figure 3.**
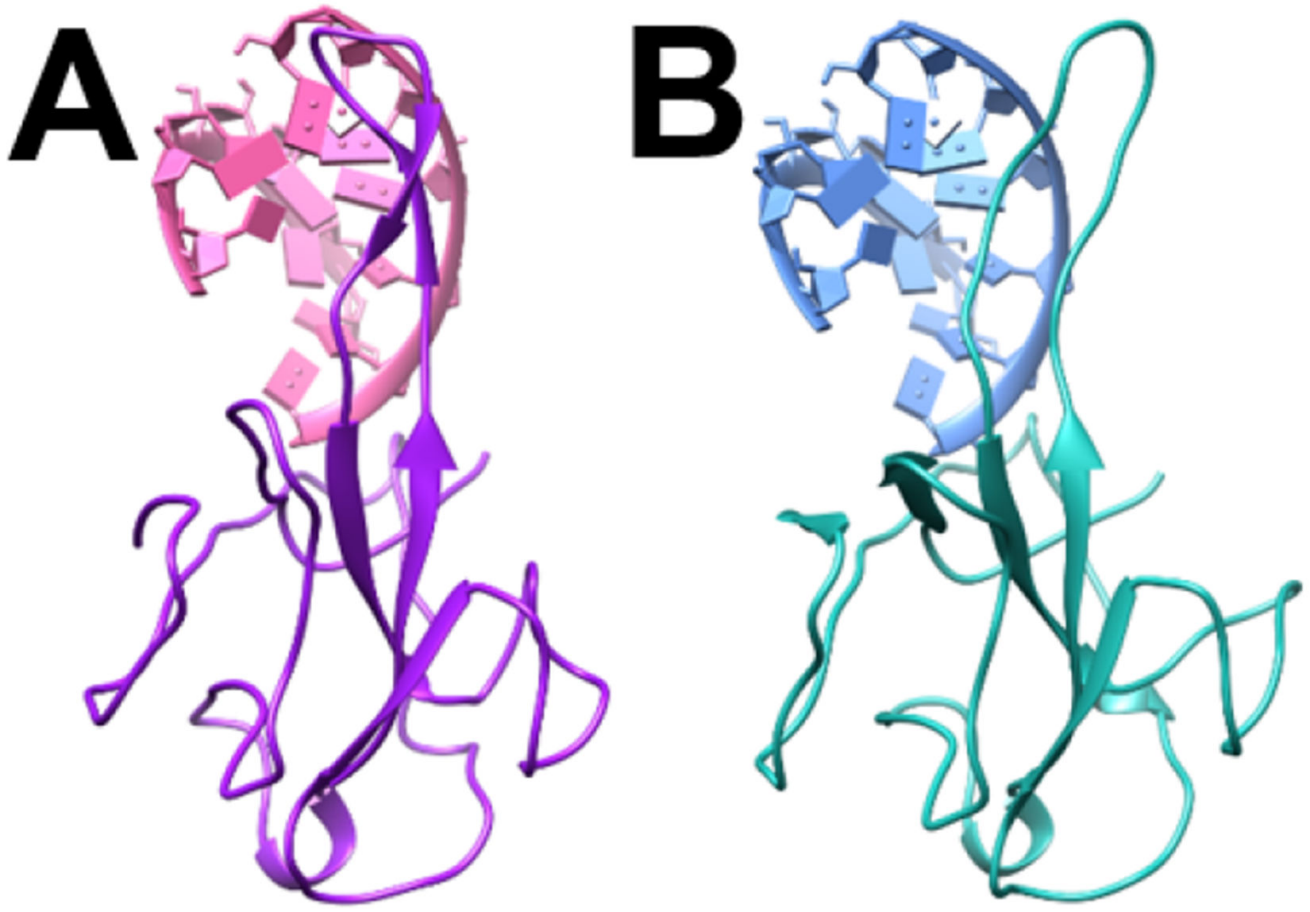
Complex structures of N protein RBDs and RNAs. (A) SARS-CoV N protein RBD (purple) bind with RNA (pink); (B) SARS-CoV-2 N protein RBD (cyan) bind with RNA (blue).

The N protein structures of SARS-CoV and SARS-CoV-2 binding with RNAs are shown in Figure 3. From the complex structures, it is obvious that the RNAs bind to the hairpin-like loop of the N proteins. The structures of SARS-CoV and SARS-CoV-2 are very similar except the hairpin-like loops. Because the hairpin-loops are much more flexible than the rest of the N protein structures. Note that the N and C terminals have been removed from both of the N proteins to obtain more stable structures, because these terminals are too flexible and have no contribution to the binding interactions. The details are shown in the methods section.

**Figure 4.**
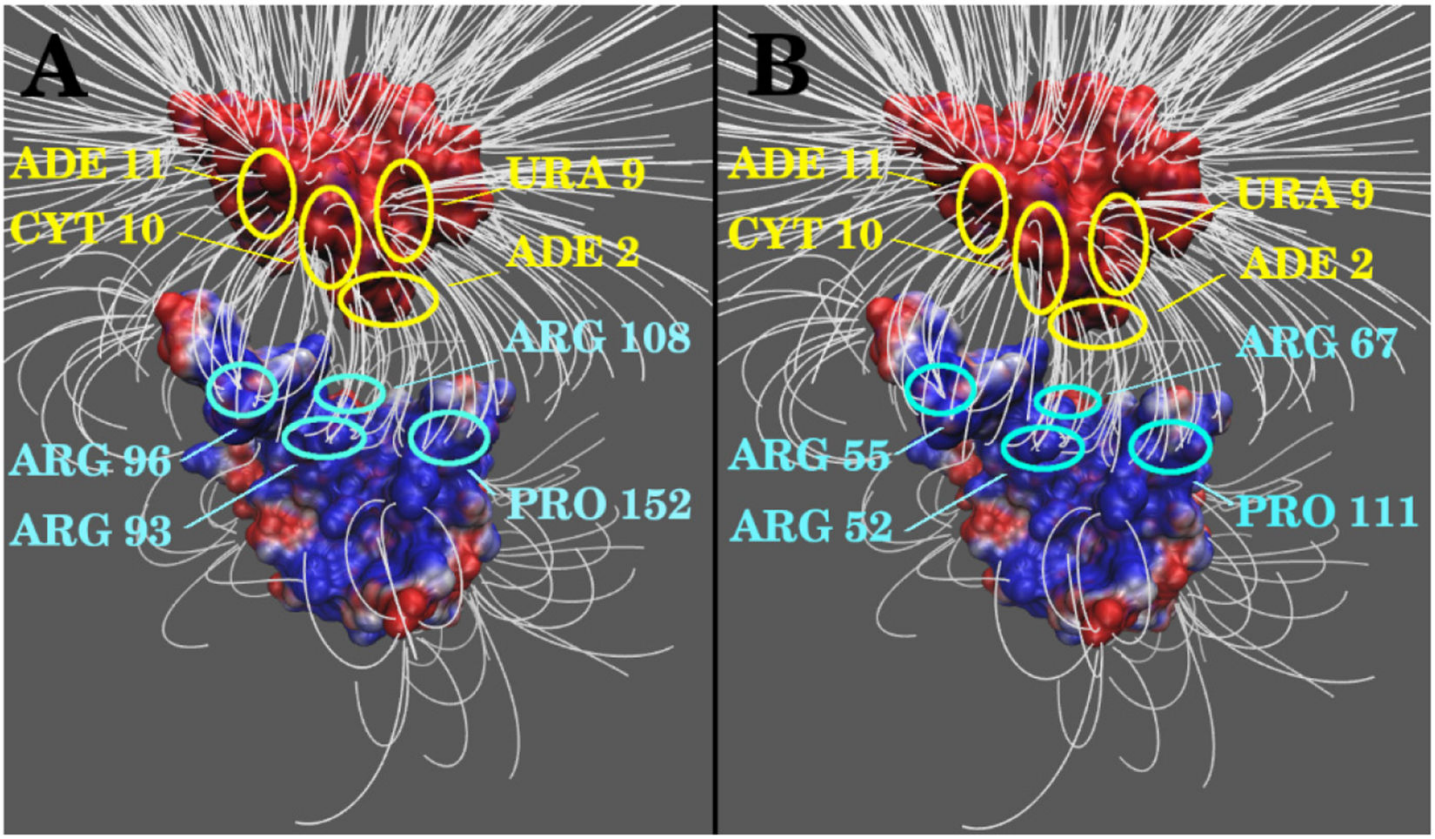
Electric field lines of complex binding domains. (A) Electric field lines of SARS-CoV N protein RBD and RNA; (B) Electric field lines of SARS-CoV-2 N protein RBD and RNA. The residues with high density of filed lines are circled and highlighted, including those on RNAs (yellow) and on N proteins (blue).

To further explore the electrostatic interactions, we calculated the electric field lines between N protein RBDs and RNAs (see Figure 4). Densities of field lines represent the strengths of electrostatic interactions. From the electric field line distributions, it is clearly shown that both N proteins of SARS-CoV and SARS-CoV-2 have strong attractive binding forces to RNAs. The residues with dense field lines on the RNA interface areas are the same, which are ADE2, URA9, CYT10 and ADE11. On the other side, the key residues generating dense field lines on N proteins of SARS-CoV and SARS-CoV-2 are also the same (note that the sequence numbers of SARS-CoV and SARS-CoV-2 have 41 residues difference). For SARS-CoV, the key residues are: ARG93, ARG96, ARG108, PRO152; for SARS-CoV-2, the corresponding identical key residues are: ARG52, ARG55, ARG67, PRO111. In each case, three out of four N proteins’ dense field lines generating residues are arginine. Also, those key residues that produce the dense electric field lines do not have any mutation from SARS-CoV to SARS-CoV-2, which means that these residues are conserved.

### 3.4. Electrostatic forces

**Figure 5.**
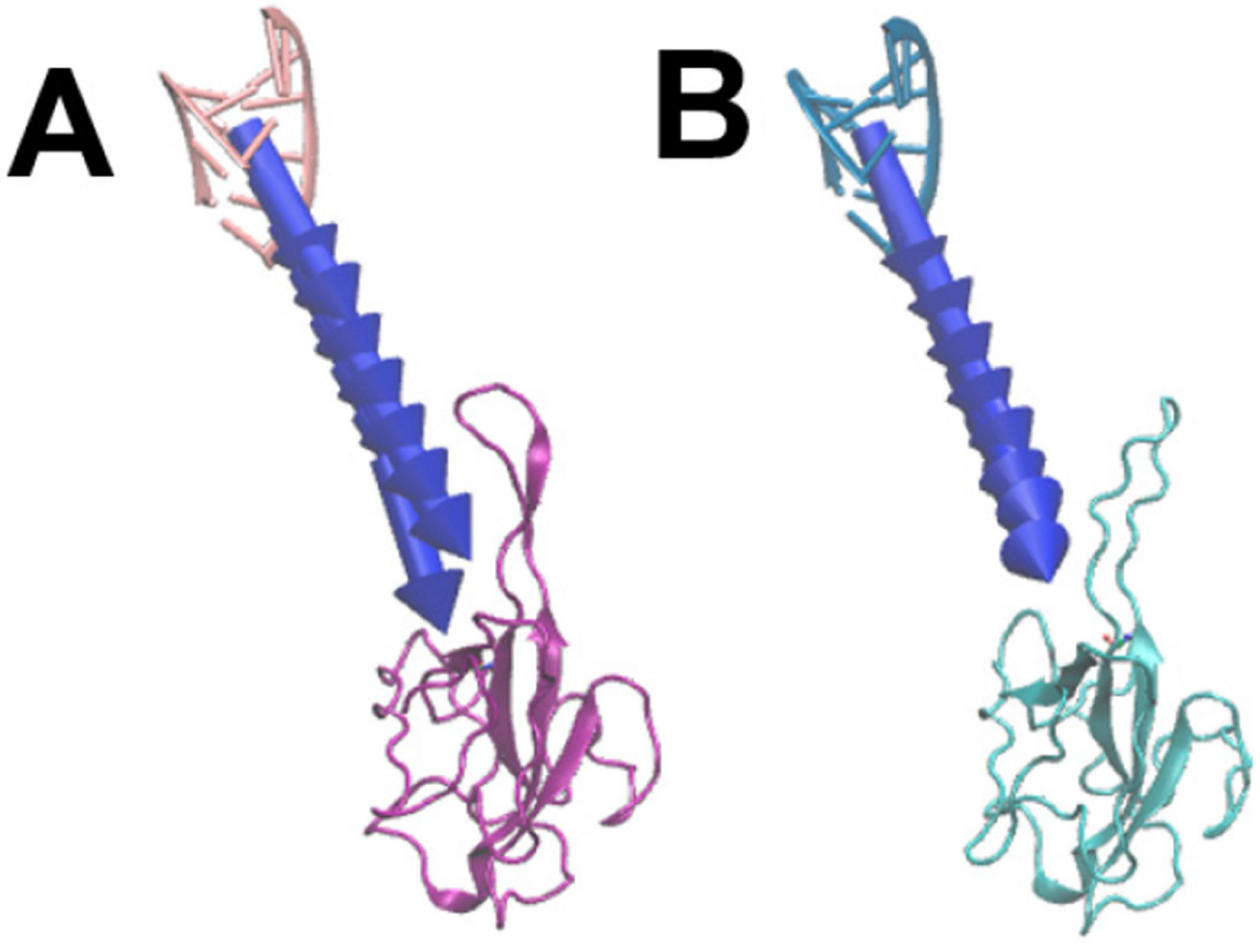
Electrostatic forces of SARS-CoV and SARS-CoV-2 N protein RBDs with RNAs at distances from 5 Å to 40 Å. (A) Electrostatic force of SARS-CoV N protein RBD (purple) and RNA(pink) at distances from 5 Å to 40 Å with a step size of 5 Å; (B) Electrostatic force of SARS-CoV-2 N protein RBD (cyan) and RNA (light blue) at distances from 5 Å to 40 Å with a step size of 5 Å.

Electrostatic forces of SARS-CoV and SARS-CoV-2 N proteins’ RBDs with RNAs at distances from 5 Å to 40 Å with a step size of 5 Å were separated by StructureMan [32] and calculated by DelPhiForce at each position (see Figure 5). The directions of the blue arrows are illustrated to show the directions of net forces between N proteins and RNAs. The arrows are normalized to the same size for better visualizations. From the figures, the electrostatic forces of N proteins attract the corresponding RNAs. It clearly showed that the directions of arrows are different by comparing SARS-CoV and SARS-CoV-2 N protein RBDs with RNAs at distance of 5 Å. It may because of the hairpin-like loop structure at the top of the N protein, which is more flexible as shown in Figure S1.

**Figure 6.**
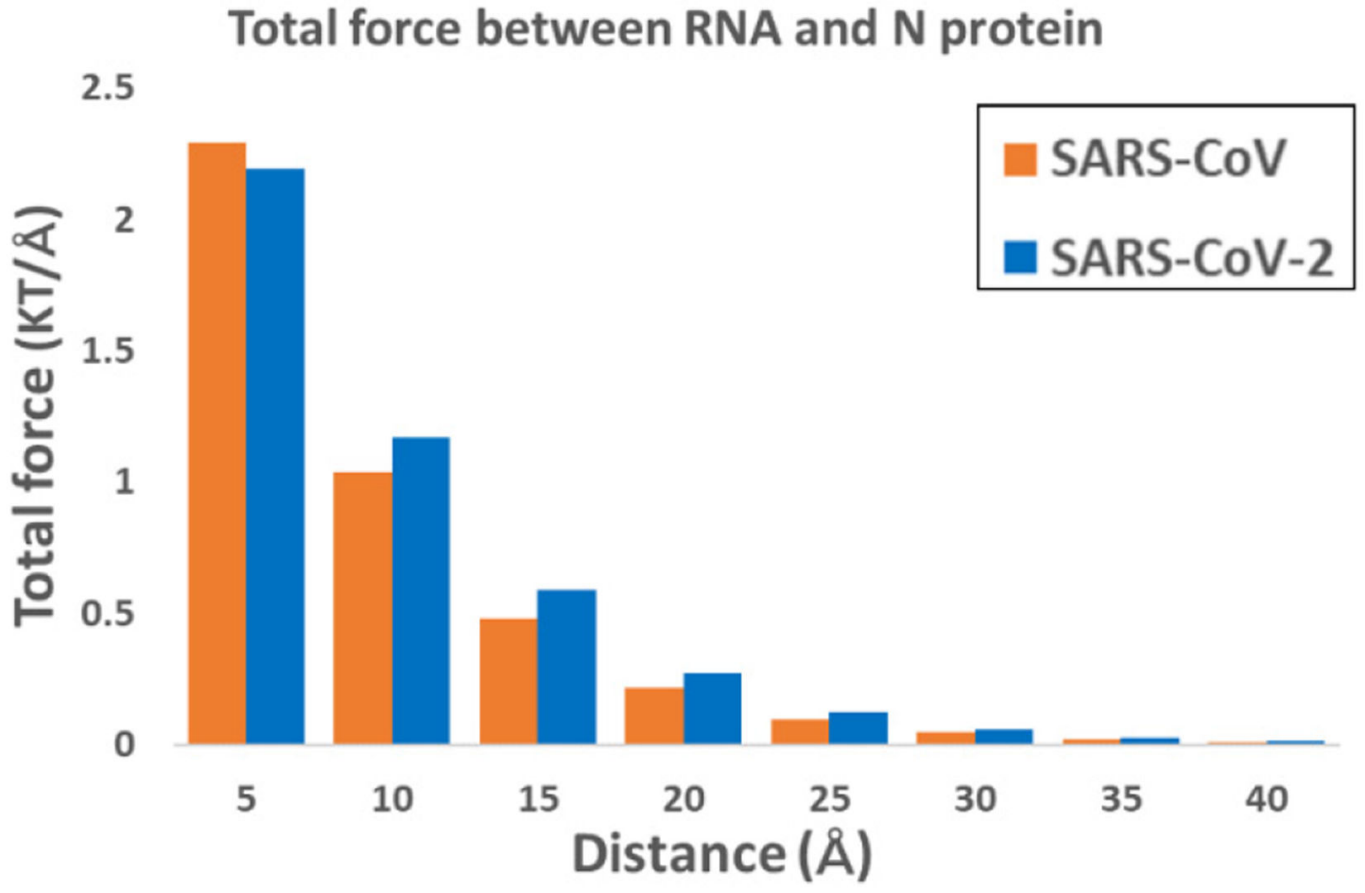
The trends of electrostatic forces of SARS-CoV and SARS-CoV-2 N proteins’ RBDs with RNAs at distances from 5 Å to 40 Å. Orange and Blue colors represent SARS-CoV and SARS-CoV-2, respectively.

While Figure 5 only focuses on the directions of the electrostatic forces, the magnitudes of these electrostatic forces of SARS-CoV and SARS-CoV-2 are shown in Figure 6. The electrostatic forces between N proteins’ RBDs and RNAs decrease as the distances increase. It is obviously shown that SARS-CoV and SARS-CoV-2 have similar electrostatic forces at different distances. SARS-CoV-2 has relatively stronger forces than SARS-CoV, except at the distance of 5 Å. Figure 6 only compares the magnitudes of the forces between SARS-CoV and SARS-CoV-2. However, the directions of the forces are also important for electrostatic forces, which is shown in Figure 5.

### 3.5. Binding energies

In protein-DNA/RNA complexes, it is common that the binding energies depend on the pH environment [29,30]. The pH-optimum is the pH at which the complex has maximal electrostatic binding energy [28]. To demonstrate the pH dependence in the binding process of N proteins and RNAs, DelPhiPKa was implemented to calculate the binding energies. It should be mentioned that the binding energies calculated using DelPhiPKa method are relative binding energies rather than absolute energies. By default, the binding energy at pH 0 is set as reference, which is 0 kcal/mol. The relative energy profile can be used to study the binding energy dependence on pHs.

**Figure 7.**
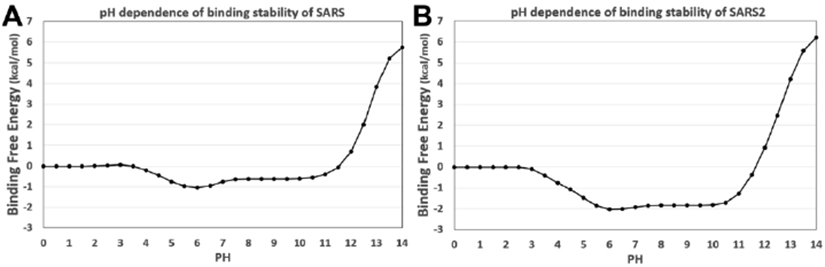
The calculated pH dependence of the binding energy between N proteins’ RBDs and RNAs. (A) The binding energy of SARS-CoV N protein RBD and RNA; (B) The binding energy of SARS-CoV-2 N protein RBD and RNA. The pH range was set from 0.0 to 14.0 with an interval size of 0.5.

The results are shown in Figure 7. From the binding energy curves, it is obvious that for both SARS-CoV and SARS-CoV-2, the binding energy is stable within a wide range of pH (from 5.5 to 10). Such pH independent binding energy phenomena were also found in some other related studies [19].

### 3.6. Folding energies

**Figure 8.**
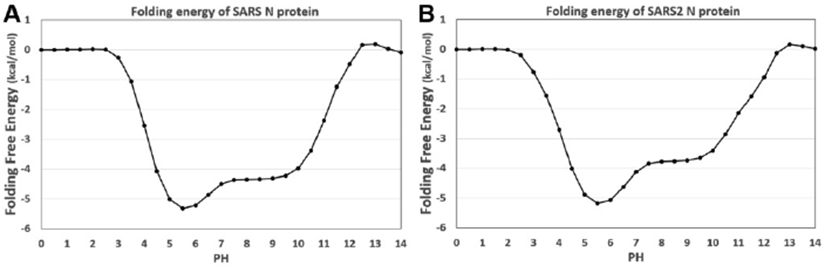
The calculated pH-dependence of the folding energy of N proteins’ RBDs and RNAs. (A) The folding energy of SARS-CoV N protein RBD and RNA; (B) The folding energy of SARS-CoV-2 N protein RBD and RNA. pH ranges from 0.0 to 14.0 with an interval of 0.5.

The net charges of SARS-CoV and SARS-CoV-2 are calculated with DelPhiPKa [26,27]. The pH range was set from 0 to 14 with an interval of 0.5. Figure 8 shows the calculated folding energies of SARS-CoV and SARS-CoV-2 at different pH values. The pH-dependence of the folding free energy demonstra that SARS-CoV and SARS-CoV-2 have the same pH-optimum value where the folding energy is the most favorable at this pH (here the pH-optimum value is 5.5). In addition, N proteins of SARS-CoV and SARS-CoV-2 have similar inverted funnel-shaped folding energy curves. These curves indicate that the pH-dependences of folding energies of SARS-CoV/SARS-CoV-2 N proteins binding with RNAs are very similar. And the combination of the folding energy and binding energy profiles demonstrates that the N proteins perform their functions best at pH 5.5.

## 4. Conclusions

Due to the sequence similarity, SARS-CoV and SARS-CoV-2 have very similar functions and structures. Each of their genes encodes four types of structural proteins, including N protein which is studied in this work. The N proteins of SARS-CoV and SARS-CoV-2 are similar in sequence and almost identical in structure. This study revealed some fundamental mechanisms of these N proteins, including their stabilities and binding strengths with RNAs at different pHs.

Multiple computational approaches were utilized in this work to investigate the N proteins. Electrostatic potential of the surfaces of N proteins show that both of the N proteins from SARS-CoV and SARS-CoV-2 have similar electrostatic potential distributions. The binding interfaces are dominantly positively charged, which results in attractive electrostatic interactions to RNAs. The electrostatic force analyses validated such attractive interactions. The binding forces between SARS-CoV N protein and RNA at different distances are similar to that of SARS-CoV-2, in both directions and magnitudes. Electric filed lines between N proteins and RNAs are also similar between SARS-CoV and SARS-CoV-2. The binding energy dependence to pHs shows that the binding of both N proteins with RNAs are stable in a wide range of pH (from pH 5.5 to 10). For folding energy dependence to pH, the optimal pH is found as 5.5 for both N proteins. This indicates that the N proteins perform their functions best in a weak acidic environment, which is perfect for theses N proteins to maintain their structures and perform functions surrounding RNAs.

## Supplementary Material

SI
