# Supplementary material for: Electrostatic features for nucleocapsid proteins of SARS-CoV and SARS-CoV-2
Source: Math Biosci Eng. Author manuscript; Available in PMC 2021 Jul 14. (PMC8279046; doi:10.3934/mbe.2021120)
Supplement: SI [file NIHMS1717636-supplement-SI.docx]

**Electrostatic features for nucleocapsid proteins of SARS-CoV and SARS-CoV-2**

Wenhan Guo^1^, Yixin Xie^1^, Alan E Lopez-Hernandez^1^, Shengjie Sun^1^, Lin Li^1,2*^

^1^ Computational Science Program, University of Texas at El Paso, El Paso, TX.

^2^ Department of Physics, University of Texas at El Paso, El Paso, TX.

*** Correspondence:**

Lin Li: [lli5@utep.edu](mailto:lli5@utep.edu)

**
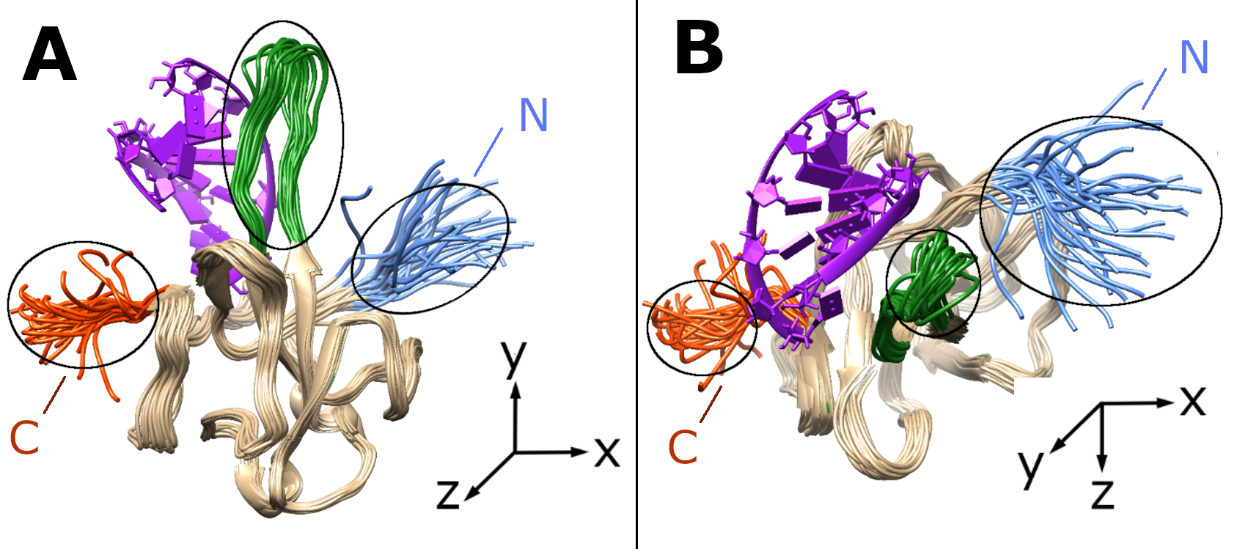
**

**Figure S1.** (A) The side view of the structure of SARS-CoV-2 N protein RBD bind with RNA; (B) The top view of the structure of SARS-CoV-2 N protein RBD bind with RNA. The N terminals are shown with blue color while the C terminals are displayed with orange color. The flexible hairpin-like structures of SARS-CoV-2 N protein RBDs are highlighted with green color.
